# The Impact of Obesity on Intraoperative Complications in Rectal Cancer

**DOI:** 10.1111/ans.70190

**Published:** 2025-05-30

**Authors:** Simon Xu, Rathin Gosavi, Yigeng Li, James Lim, T. C. Nguyen, William Teoh, Geraldine Ooi, Vignesh Narasimhan

**Affiliations:** ^1^ Department of Colorectal Surgery Monash Health Melbourne Australia; ^2^ Department of Upper GI Surgery Monash Health Melbourne Australia; ^3^ Department of Colorectal Surgery Cabrini Health Melbourne Australia; ^4^ Department of Surgery School of Clinical Sciences at Monash Health, Monash University Melbourne Australia

**Keywords:** CLASSIntra, intraoperative adverse events, laparoscopic surgery, obesity, rectal cancer surgery, surgical outcomes

## Abstract

**Background:**

Obesity is a growing global health concern and poses significant challenges in rectal cancer surgery. Excess visceral fat can obscure surgical landmarks, complicate dissection, and increase the risk of intraoperative adverse events (iAEs). Despite these recognized difficulties, there is limited objective data quantifying the impact of obesity on intraoperative complications. This study utilizes the CLASSIntra classification system to assess the incidence and severity of iAEs in patients with obesity (BMI ≥ 30 kg/m^2^) undergoing rectal cancer resection.

**Methods:**

This retrospective cohort study reviewed patients undergoing rectal cancer resection between January 2014 and December 2023. Patients were stratified into groups by BMI (obese (BMI≥ 30 kg/m^2^) versus non‐obese (BMI < 30 kg/m^2^)). The primary outcome was the incidence and severity of iAEs, graded using the CLASSIntra system. Secondary outcomes included conversion to open surgery, postoperative complications, ICU admissions, and overall length of stay.

**Results:**

There were 350 patients included (112 obese, 238 non‐obese). There were significantly more iAEs in the obese group (40% vs. 26%, *p* = 0.010). Obesity was an independent predictor of intraoperative complications (OR 1.92, *p* = 0.010). Conversion to open surgery (27% vs. 40%, OR 2.30, *p* = 0.0100) and ICU readmission (50% vs. 31%, OR 2.35, *p* = 0.003) were significantly more common in patients with obesity. There were no significant differences in postoperative complication rates and hospital length of stay between groups.

**Conclusion:**

Obesity leads to a higher risk of intraoperative complications in rectal cancer surgery, increased conversion rates, and greater ICU resource utilisation. These findings highlight the technical challenges of rectal surgery in obese patients and emphasize the need for tailored preoperative planning, prehabilitation, and intraoperative strategies. Despite these intraoperative difficulties, structured postoperative care appears to mitigate differences in postoperative outcomes. Further research should explore preoperative interventions, such as weight optimization programs, to improve surgical outcomes in this high‐risk population.

## Graphical abstract

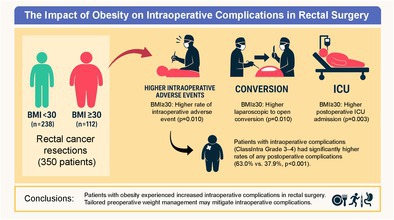

## 1 Introduction

Obesity is a growing global health concern, with over 890 million adults affected worldwide [1]. In Australia, 31% of adults are classified as obese (BMI ≥ 30 kg/m^2^) [2], a trend linked to the metabolic syndrome and increasingly sedentary lifestyles [3]. Obesity poses particular challenges in surgical care, such as in rectal cancer surgery. It complicates pelvic surgery due to increased visceral adiposity limiting surgical exposure [4, 5] with altering tissue planes, all of which potentially contribute to a higher risk of intraoperative adverse events (iAEs) [6]. While the technical difficulties associated with obesity in rectal cancer surgery are widely acknowledged, objective data quantifying iAEs in this patient group remain limited.

The CLASSIntra classification, validated in 2020 [7], provides a standardized system for grading intraoperative surgical and anesthesia‐related complications, ranging from minor bleeding (Grade I) to life‐threatening events (Grade V) (Table 1). The classification system provides an objective framework to assess intraoperative adverse events, ensuring consistency in reporting and reducing variability. It serves as an effective communication and handover tool, facilitating clear and structured discussions amongst surgical teams, anaesthetists, and postoperative care providers [8]. By enabling hospitals to track and compare complication rates, it supports intraoperative quality improvement efforts and contributes to better patient safety. Additionally, its structured approach also strengthens research and clinical trials by ensuring more reliable comparisons of surgical outcomes.

**TABLE 1:** CLASSIntra classification of intraoperative complications.

Grade 0	Ideal operative course
Grade 1	Deviation from the ideal operative course without the need for additional intervention. May result in no or mild symptoms
	Grade 1 examples: Bleeding that is self‐limiting or requiring only routine cautery. Minimal serosal injury not requiring additional treatment. Small burn of skin with cautery. Arrhythmias without relevance.
Grade 2	Deviation from the ideal operative course with the need for additional treatment or minor intervention. May result in moderate symptoms, not resulting in permanent disability or threat to life.
	Grade 2 examples: Bleeding requiring ligation or use of tranexamic acid. Non‐transmural intestinal lesion requiring suturing. Moderate burn requiring non‐invasive wound care. Arrhythmia requiring administration of antiarrhythmic drugs without hemodynamic effect.
Grade 3	Deviation from the ideal intraoperative course with the need for additional moderate treatment or intervention. May result in severe symptoms, potentially leading to permanent disability and potentially life threatening.
	Grade 3 examples: Bleeding resulting in transient hemodynamic instability. Managed with ligation, suture or blood transfusion. Transmural intestinal injury requiring segment resection. Severe burn requiring surgical debridement. Arrhythmias requiring antiarrhythmic drug administration with transient hemodynamic effect.
Grade 4	Deviation from the ideal intraoperative course with the need for major and urgent treatment or intervention. Life threatening or leading to permanent disability.
	Grade 4 examples: Bleeding resulting in a threat to life, requiring massive transfusion, splenectomy or admission to the intensive care unit Injury or a central vessel requiring extended intestinal resection Life threatening burn injury leading to fire or requiring admission to the intensive care unit Arrhythmia requiring electro‐conversion, defibrillation or admission to the intensive care unit.
Grade 5	Death

In this study, we primarily aimed to identify the impact of obesity on the incidence and severity of intraoperative adverse events in patients undergoing rectal cancer resection, using the CLASSIntra classification system. Secondary objectives include assessing conversion rates, postoperative morbidity, and resource utilization. By investigating the impact of BMI on intraoperative complications, this analysis aims to improve preoperative counseling, surgical planning, and institutional resource allocation.

## 2 Methods

### 2.1 Study Design and Setting

This retrospective cohort study included patients who underwent rectal cancer resection at a single metropolitan referral centre over a nine‐year period (January 2014–December 2023). Data were obtained from the institution's prospectively maintained colorectal surgery database and cross‐referenced with electronic medical records (EMRs) to ensure completeness.

### 2.2 Study Population and Eligibility Criteria

Patients aged 18 years or older who underwent elective open or laparoscopic rectal resection for histologically confirmed adenocarcinoma were included. Patients were excluded if surgery was performed for benign pathology, recurrent rectal cancer, or metastatic disease requiring palliative resection.

Patients were stratified into two cohorts based on body mass index (BMI): an obese group (BMI ≥ 30 kg/m^2^, per WHO criteria for Class I–III obesity) and a non‐obese group (BMI < 30 kg/m^2^). BMI values were calculated using preoperative height and weight measurements documented in anesthesia records.

### 2.3 Data Collection and Variables

Data were extracted from EMRs, operative reports, and anesthesia records. Demographic variables included age, sex, smoking status, and comorbidities such as ischemic heart disease (IHD), diabetes mellitus, and chronic obstructive pulmonary disease (COPD). Operative details were obtained from operation reports, including surgical approach (laparoscopic vs. open), conversion to open surgery, estimated blood loss (EBL), and total operative time.

### 2.4 Outcome Measures

The primary outcome was the incidence and severity of iAEs, classified using the CLASSIntra system. Secondary outcomes included conversion to open surgery, postoperative complications (graded by the Clavien‐Dindo classification within 30 days), intensive care unit (ICU) admission, and hospital length of stay (LOS).

### 2.5 CLASSIntra Grading Protocol

Two independent reviewers assigned CLASSIntra grades based on operative and anesthesia reports. Discrepancies were resolved by review of the notes by the senior author. CLASSIntra grading [7] was defined as follows: Grade I represented minor deviations (e.g., minor bleeding controlled with electrocautery), Grade II indicated unplanned but manageable interventions (e.g., suture repair of serosal tears), Grades III–IV reflected more significant complications (e.g., bowel injury requiring resection or anastomotic revision), and Grade V signified life‐threatening events (e.g., massive hemorrhage or cardiac arrest). ClassIntra Grading is summarized in Table 1.

## 3 Statistical Analysis

Continuous variables were reported as mean ± standard deviation (SD) for normally distributed data or median with interquartile range (IQR) for skewed data. Categorical variables were summarized as frequencies and percentages. Group comparisons used Student's *t*‐test or Mann–Whitney *U*‐test for continuous variables and Pearson's chi‐squared or Fisher's exact test for categorical variables. Multivariable logistic and linear regression models adjusted for potential confounders, including age, sex, American Society of Anaesthesiologists (ASA) physical status score, comorbidities, smoking status, presence of ischemic heart disease, diabetes, use of antiplatelet agents, neoadjuvant therapy (NAT) and surgical approach (laparoscopic vs. open vs. conversion to open). A *p*‐value of < 0.05 was considered statistically significant. Analyses were performed using Stata version 17.0 (StataCorp 2023).

## 4 Results

### 4.1 Patient Characteristics

A total of 350 patients were included. The mean age of the population was 63 +/− 13, with 66% being male and a median ASA score of 2.

There were 238 patients (68%) in the BMI < 30 group and 112 (32%) in the BMI ≥ 30 group. Obese patients were more likely to have ischemic heart disease (*p* = 0.045), but other comorbidities were comparable between groups. There were no significant differences in age, BMI, ASA score, other comorbidities, surgical approach, or type of operation. Full demographics are summarized in Table 2.

**TABLE 2:** Baseline characteristics of participants.

Variables	Total (*n* = 350)	BMI in two categories (kg/m^2^)		
		< 30 (*n* = 238)	>/= 30 (*n* = 112)	*p*
Age, years (mean +/−sd)	62.8 +/−12.7	63.3 +/−13.1	61.6 +/−12.0	0.24
Sex (male, %)	230 (65)	162 (68)	68 (61)	0.18
Comorbidities (yes, %)				
Smoking	155 (44)	109 (46)	46 (41)	0.41
Ischaemic heart disease	47 (13)	26 (11)	21 (19)	0.05
Diabetes	66 (19)	45 (19)	21 (19)	0.97
Use of antiplatelet therapy	40 (11)	24 (10)	16 (14)	0.25
Neoadjuvant therapy (yes %)	200 (68)	137 (67)	63 (70)	0.67
Surgical approach (number, %)				0.06
Laparoscopic	181 (51)	133 (56)	48 (43)	
Open	87 (24)	56 (23)	31 (25)	
Conversion from lap to open	82 (23)	49 (21)	33 (29)	
ASA (*n*, %)				0.16
Median (IQR)	2 (2–3)	2 (2–3)	3 (2–3)	
1	21 (6)	15 (6)	6 (5)	
2	174 (50)	127 (53)	47 (42)	
3	147 (42)	92 (39)	55 (49)	
4	8 (2)	4 (2)	4 (4)	
5	0 (0)	0 (0)	0 (0)	
Operation type (*n*, %)				0.48
APR/proctocolectomy	109 (31)	76 (32)	33 (29)	
ULAR	170 (49)	118 (50)	52 (46)	
Low anterior resection	71 (20)	44 (18)	27 (24)	

Abbreviations: ASA—American Society of Anaesthesiology classification; APR—abdominoperineal resection; BMI—body mass index; IQR—interquartile rante; sd—standard deviation; ULAR—ultralow anterior resection.

### 4.2 Intraoperative Complications

**FIGURE 1:**
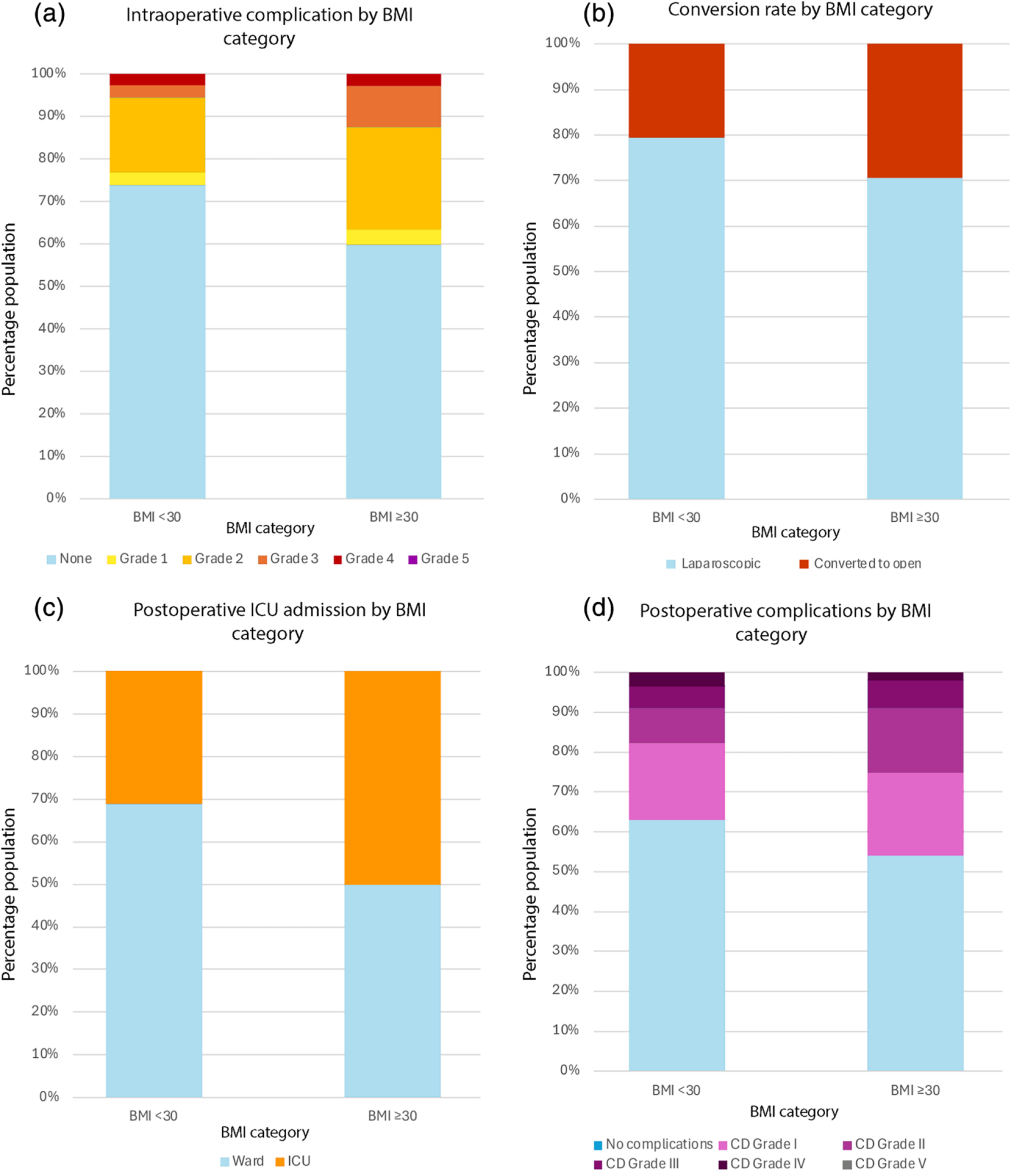
Comparison of outcomes between patients with BMI < 30 and BMI ≥ 30 undergoing rectal cancer resection. (a) Intraoperative complications‐patients with BMI ≥ 30 had a significantly higher incidence of intraoperative complications (40%) compared to those with BMI < 30 (26%, *p* = 0.010), (b) conversion rate—conversion to open surgery was more frequent in obese patients (40% vs. 27%, adjusted OR 2.30, *p* = 0.010), (c) postoperative ICU admission—obese patients required ICU admission more often (50% vs. 31%, adjusted OR 2.35, *p* = 0.003), (d) postoperative complications—while overall postoperative complications were numerically higher in obese patients (46% vs. 37%), this difference was not statistically significant (*p* = 0.11).

A total of 107 patients (31%) experienced an intraoperative complication (Table 3). This was significantly higher in patients with BMI ≥ 30 kg/m^2^ compared to those with BMI < 30 kg/m^2^ (40% vs. 26%, *p* = 0.010) (Figure 1). After adjusting for potential confounders including neoadjuvant therapy, obesity remained an independent predictor of intraoperative complications with an odds ratio (OR) of 2.01 (95% CI 1.16–3.51, *p* = 0.010). Obese patients were less likely to have an uncomplicated intraoperative course, with only 60% having no complications compared to 74% of non‐obese patients. While there was no statistically significant difference in overall postoperative complication rates, obese patients were more likely to experience higher‐grade complications, with a greater proportion experiencing Clavien‐Dindo Grade III and IV events compared to non‐obese patients (13% vs. 6%).

**TABLE 3:** Comparison of primary and secondary outcomes based on BMI of patients, unadjusted and adjusted.

Variables	Total (*n* = 350)	BMI in two categories (kg/m^2^)					
		< 30 (*n* = 238)	>/= 30 (*n* = 112)	OR unadjusted (95% CI)	*p* (unadjusted)	OR adjusted^a^ (95% CI)	*p* (adjusted^a^)
Any intra‐op Complications (yes, %)	107 (31)	62 (26)	45 (40)	1.91 (1.18–3.07)	0.01	2.01 (1.16–3.51)	0.01
Grade 0	243 (69)	176 (74)	67 (60)				
Grade 1	11 (3)	7 (3)	4 (4)				
Grade 2	69 (20)	42 (18)	27 (24)				
Grade 3	18 (5)	7 (3)	11 (10)				
Grade 4	9 (3)	6 (3)	3 (3)				
Grade 5	0 (0)	0 (0)	0 (0)				
Any post‐op complications (yes, %)	139 (40)	88 (37)	51 (46)	1.45 (0.92–2.29)	0.11	1.61 (0.96–2.73)	0.08
No complications	210 (60)	150 (63)	60 (54)				
CD Grade I	69 (20)	46 (19)	23 (21)				
CD Grade II	39 (11)	21 (9)	18 (16)				
CD Grade III	21 (6)	13 (5)	8 (7)				
CD Grade IV	10 (3)	8 (3)	2 (2)				
CD Grade V	0 (0)	0 (0)	0 (0)				
CD Grade III or greater (yes, %)	31 (9)	21 (8)	10 (9)	1.02 (0.46–2.25)	0.96	1.48 (0.62–3.54)	0.38
Conversion rate (yes, %)	82 (31)	49 (27)	33 (40)	1.87 (1.08–3.24)	0.03	2.76 (1.40–5.45)	0.003
ICU admission (yes, %)	130 (37)	74 (31)	56 (50)	2.17 (1.37–3.44)	0.001	2.44 (1.38–4.32)	0.002
Length of ICU admission in days (mean+/−sd)	2.7+/−3.1	2.9+/−3.2	2.3+/−2.3		0.03		0.10
Length of hospital day in days (mean +/− sd)	11.6 +/−9.9	11.4 +/− 10.4	12.0 +/−8.8		0.60		0.50

Abbreviations: BMI—body mass index; CD—Clavien Dindo classification of postoperative complications; CI—confidence interval; ICU—intensive care unit; OR—odds ratio; sd—standard deviation.

^a^ Adjusted for age, sex, comorbidities (smoking, ischaemic heart disease, diabetes, use of antiplatelet agents), neoadjuvant therapy, type of operation, ASA, and surgical entry (except for conversion rate).

### 4.3 Secondary Outcomes

Secondary outcome measures are shown in Table 3.

The overall conversion rate for the entire population was 31%. This was significantly greater in the BMI ≥ 30 group compared to the BMI < 30 group (40% vs. 27%, *p* = 0.030). On multivariate analysis, the difference was more pronounced, with an adjusted OR of 2.76 (95% CI 1.40–5.45, *p* = 0.003).

The overall post‐operative ICU admission rate was 37% (*n* = 130). Patients with obesity had a significantly higher likelihood of requiring ICU admission (50% vs. 31%, *p* = 0.001). After multivariate analysis, obesity remained an independent predictor of ICU admission (OR 2.44, 95% CI 1.38–4.32, *p* = 0.002).

Postoperative complications occurred in 139 (40%) of the study population, with 31 (9%) experiencing Clavien‐Dindo (CD) Grade 3 or greater complications. There were no significant differences in the rates of overall postoperative complications (46% vs. 37%, *p* = 0.11) or Grade 3 or greater complications (9% vs. 8%, *p* = 0.96).

**FIGURE 2:**
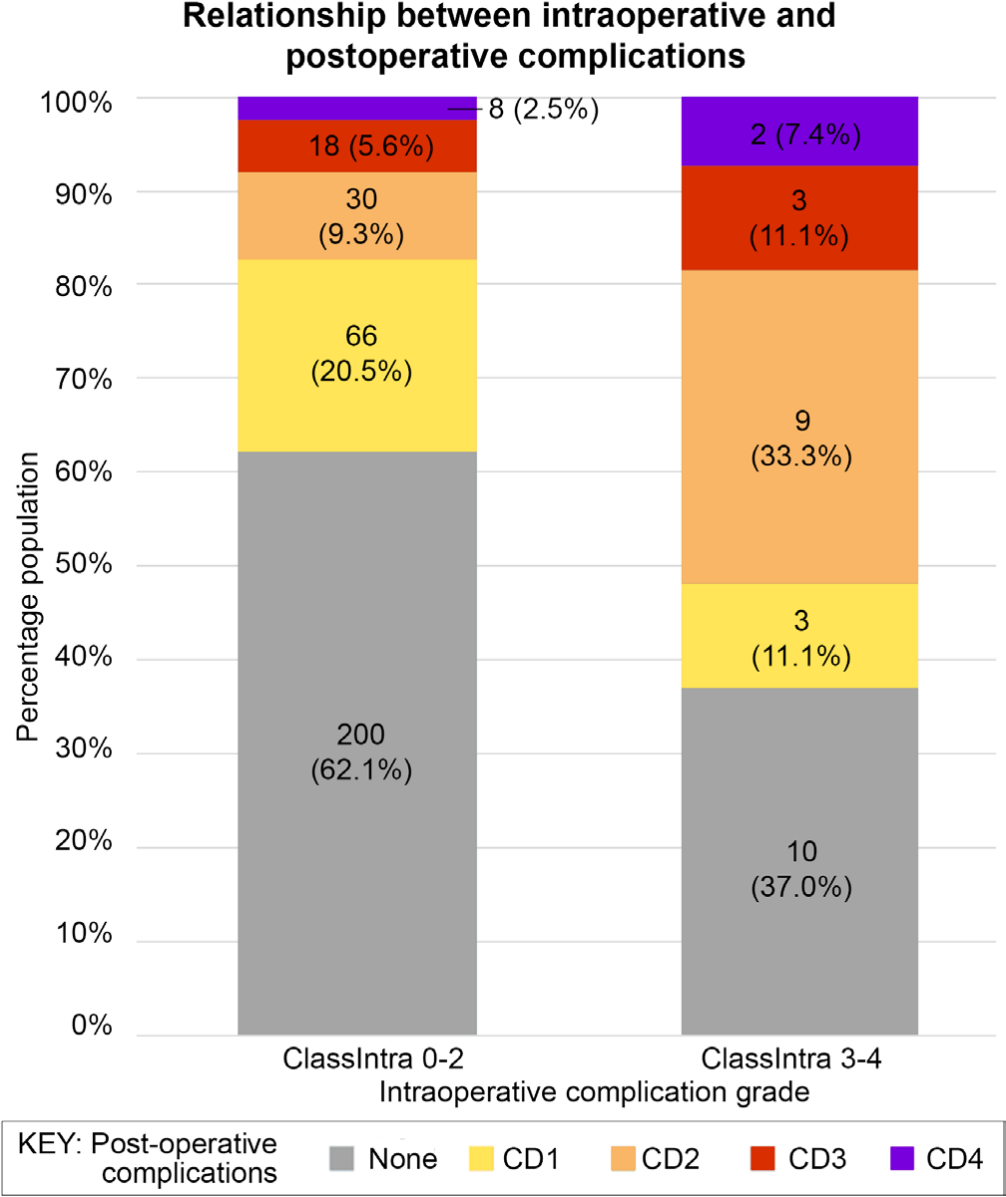
Relationship between intraoperative complications and subsequent postoperative complications. Patients experiencing intraoperative complications (ClassIntra Grade 3–4) had significantly higher rates of any postoperative complications (63.0% vs. 37.9%, *p* < 0.001).

Patients who experienced higher grades of intraoperative complications (ClassIntra 3–4, *n* = 27) had significantly higher rates of any postoperative complications (63% vs. 38%, *p* < 0.001) (Figure 2). There were no significant differences in serious postoperative complications (CD 3+) between the two groups (9% vs. 8%, *p* = 0.96).

The average length of hospital stay was 11.6 days, with no significant differences between BMI groups (*p* = 0.50).

The influence of neoadjuvant therapy on intraoperative and postoperative outcomes was assessed in Table 4. There were no significant differences in intraoperative complication rates between patients who received neoadjuvant therapy and those who did not (30% vs. 30%, *p* = 0.99). Similarly, postoperative complication rates (42% vs. 44%, *p* = 0.70), conversion rates (33% vs. 35%, *p* = 0.79), and ICU admissions (42% vs. 33%, *p* = 0.16) were comparable between the two groups. After adjusting for potential confounders, neoadjuvant therapy did not emerge as an independent predictor for intraoperative or postoperative complications, conversion to open surgery, or ICU admission.

**TABLE 4:** Assessing neoadjuvant therapy as an independent factor in primary and secondary outcome.

Variable	No neoadjuvant therapy (*n* = 93)	Neoadjuvant therapy (*n* = 200)	OD (95% CI)	*p*	Adjusted OD (95% CI)^a^	Adjusted *p*
Any intra‐op complications (yes, %)	28 (30)	60 (30)	0.99 (0.58–1.70)	0.99	0.81 (0.43–1.51)	0.51
Any post‐op complications (yes, %)	41 (44)	83 (42)	0.91 (0.55–1.49)	0.70	0.78 (0.44–1.38)	0.39
Conversion rate (yes, %)	26 (35)	48 (33)	0.92 (0.51–1.66)	0.79	0.98 (0.49–1.93)	0.95
ICU admission (yes, %)	31 (33)	84 (42)	1.45 (0.87–2.42)	0.16	1.33 (0.68–2.61)	0.41
Length of ICU admission in days (mean+/−sd)	2.5 +/−2.4	2.4 +/− 2.2		0.34		0.99
Length of hospital day in days (mean +/− sd)	11.8 +/− 8.7	12.3 +/− 12.4		0.70		0.24

Abbreviations: BMI—body mass index; CD—Clavien Dindo classification of postoperative complications; CI—confidence interval; ICU—intensive care unit; OR—odds ratio; sd—standard deviation.

^a^ Adjusted for age, sex, comorbidities (smoking, ischaemic heart disease, diabetes, use of antiplatelet agents), BMI, type of operation, ASA, and surgical entry (except for conversion rate).

## 5 Discussion

This study highlights the complex interplay between obesity and surgical outcomes in rectal cancer resections. Obese patients experienced significantly higher intraoperative complication rates, a greater need for conversion to open surgery, and increased ICU admissions. Intraoperative complications were associated with higher rates of postoperative complications. These findings suggest that obesity not only complicates technical aspects of surgery but also places greater demand on perioperative resources.

One of the key challenges in obese patients is limited pelvic exposure. Excess visceral adiposity makes it challenging to visualize and manipulate critical structures, increasing the risk of inadvertent injury to adjacent organs. Additionally, the altered tissue planes and increased mesorectal fat can lead to more challenging dissections [9], increasing operative time [10] and blood loss [11]. Moreover, obesity‐associated chronic inflammation and endothelial dysfunction may impair tissue healing and increase susceptibility to hemorrhage [12]. This is compounded by the physiological strain imposed by obesity, which can contribute to prolonged postoperative recovery and greater reliance on intensive care support [13].

These factors collectively compromise technical precision, necessitating heightened vigilance and judicious conversion to open surgery when warranted. While these challenges are partly shared with other abdominal surgeries [14, 15], the confined pelvic space and proximity to critical structures in rectal resections magnify the risks unique to this procedure [16, 17]. Surgeons should anticipate these difficulties preoperatively and consider patient selection carefully when planning minimally invasive procedures.

Interestingly, while intraoperative complications and ICU admission rates were significantly higher in obese patients, postoperative complication rates and overall length of hospital stay were not statistically different between BMI groups. This finding suggests that while obesity contributes to intraoperative technical difficulties, well‐structured postoperative care protocols may help mitigate its impact on longer‐term recovery. However, it is possible that differences in postoperative outcomes might emerge in larger cohorts or with longer follow‐up periods.

Our study found that NAT did not significantly impact intraoperative or postoperative complications, conversion rates, or ICU admissions, suggesting that while NAT is crucial for tumor downstaging, its effect on perioperative morbidity is limited. This aligns with prior research showing inconsistent effects of NAT on surgical complexity, with some studies reporting increased fibrosis and technical difficulty [18, 19], while others, including ours, found no significant increase in intraoperative adverse events [20]. These findings support the notion that NAT does not inherently elevate surgical risk, affirming its continued oncologic use without concern for increased perioperative complications.

Preoperative weight loss may mitigate obesity‐related intraoperative challenges in rectal cancer surgery by addressing both technical and physiological risk factors [21]. By reducing visceral adiposity, weight optimisation could enhance surgical exposure, facilitate dissection in a narrow pelvic cavity [22], and potentially decrease conversion rates. Additionally, improved metabolic profiles associated with weight reduction may help ameliorate obesity‐related comorbidities such as diabetes and cardiopulmonary disease, which further contribute to surgical risk. Beyond intraoperative benefits, preoperative weight loss could also improve postoperative outcomes by reducing infection rates, enhancing wound healing, and decreasing the need for intensive perioperative support [23]. While this study did not directly assess preoperative weight loss interventions, the observed association between obesity and intraoperative complications suggests that structured weight management programs could serve as a modifiable risk factor to improve both surgical feasibility and patient outcomes.

The integration of robotic‐assisted surgery, as evidenced by the ROLARR trial, offers potential technical advantages in rectal cancer resections, particularly for high‐risk populations such as obese patients. While the trial demonstrated no significant difference in overall postoperative outcomes between robotic and laparoscopic approaches, robotic surgery was associated with numerically lower conversion rates to open surgery (8.1% vs. 12.2%), particularly among male patients and those with low rectal tumors (OR 0.46, 95% CI 0.21–0.99) [24]. This aligns with findings from the ALaCaRT trial, which identified obesity as an independent predictor of technically challenging resections due to limited pelvic exposure and altered tissue planes [25]. Robotic platforms, with their enhanced articulation, stable visualization, and ergonomic benefits, may mitigate some of these challenges, potentially reducing intraoperative strain and conversion rates. However, the broader applicability of robotics remains constrained by cost, learning curves, and institutional resources, highlighting the need for cautious patient selection and surgeon expertise in optimizing outcomes for obese patients.

This study has a few inherent limitations. The single center retrospective nature introduces risks of selection bias and incomplete data capture. Reliance on electronic medical records (EMRs) and operative reports may lead to underreporting of intraoperative events or inconsistencies in documentation in operation reports. Retrospective analyses cannot establish causality and are inherently limited by confounding variables that were not measured or adjusted for, such as variations in surgical technique over time. Furthermore, the cohort included 350 patients, with only 112 in the obese group. This limited sample size may reduce statistical power to detect differences in rare outcomes or subtle postoperative morbidity trends. Larger, multi‐center prospective studies are needed to validate these findings.

This study highlights the significant impact of obesity on intraoperative complexity in rectal cancer surgery. Patients with a BMI ≥ 30 kg/m^2^ faced almost double the risk of iAEs, higher conversion rates, and greater ICU admission requirements. These findings underscore the challenges of minimally invasive techniques in this population and the need for tailored operative and perioperative strategies. While intraoperative difficulties were more pronounced, structured postoperative care helped mitigate disparities in morbidity and hospital stay. Future research should explore preoperative interventions, such as weight management programs, and intraoperative strategies or use of a robot platform to optimize outcomes for obese patients undergoing rectal cancer surgery. A multidisciplinary approach remains key in addressing these surgical challenges effectively.

## Ethics Statement

Ethics approval was granted by Monash Health Human Research Ethics Committee (Ref No. RES‐23‐0000‐918Q).
